# The T Cell Response to the Contact Sensitizer Paraphenylenediamine Is Characterized by a Polyclonal Diverse Repertoire of Antigen-Specific Receptors

**DOI:** 10.3389/fimmu.2017.00162

**Published:** 2017-02-16

**Authors:** Theres Oakes, Amy Lee Popple, Jason Williams, Katharine Best, James M. Heather, Mazlina Ismail, Gavin Maxwell, Nichola Gellatly, Rebecca J. Dearman, Ian Kimber, Benny Chain

**Affiliations:** ^1^Division of Infection and Immunity, UCL, London, UK; ^2^Faculty of Life Sciences, University of Manchester, Manchester, UK; ^3^Contact Dermatitis Investigation Unit, Salford Royal NHS Foundation Trust, Manchester, UK; ^4^Unilever Safety and Environmental Assurance Centre, Colworth Science Park, Sharnbrook, UK

**Keywords:** allergic contact dermatitis, T cell receptor repertoire, paraphenylenediamine, T cell immunity, skin sensitizer

## Abstract

Paraphenylenediamine (PPD) is a common component of hair dyes and black henna tattoos and can cause skin sensitization and allergic contact dermatitis (ACD). The cutaneous inflammatory reaction associated with ACD is driven by both CD4+ and CD8+ T cells. However, the characteristics of such responses with respect to clonal breadth and magnitude are poorly defined. In this study, we have characterized the *in vitro* recall response of peripheral blood T cells prepared from PPD-allergic individuals to a PPD–human serum albumin (HSA) conjugate (PPD–HSA). Quantitative high throughput sequencing was used to characterize the changes in the repertoire of T cell receptor (TCR) α and β genes after exposure to antigen *in vitro*. The PPD conjugate induced expansion of T cells carrying selected TCRs, with around 800 sequences (around 1%) being 8 or more times as abundant after culture than before. The expanded sequences showed strong skewing of V and J usage, consistent with an antigen-driven clonal expansion. The complementarity-determining region 3 sequences of the expanded TCRs could be grouped into several families of related amino acid sequence, but the overall diversity of the expanded sample was not much less than that of a random sample of the same size. The results suggest a model in which PPD–HSA conjugate stimulates a broad diversity of TCRs, with a wide range of stimulation strengths, which manifest as different degrees of *in vitro* expansion.

## Introduction

Allergic contact dermatitis (ACD) is a T cell-mediated skin disease. It results from the acquisition of skin sensitization to chemicals (metal ions and organic chemicals) encountered at skin surfaces (1). ACD is the most common manifestation of immunotoxicity in humans and can be caused by a wide array of chemicals including nickel (jewelry), fragrances, preservatives, rubber (gloves), dye (hair colorants), adhesives of various kinds, and topical medications, including antibiotics. Little is known about what drives variation in the human immune response to sensitizing chemicals, and consequently a better understanding of the molecular and cellular events that underlie skin sensitization would help improve the safety assessment of the sensitizing properties of new chemicals (2), as well as potentially leading to improved therapeutic strategies. Both CD4+ and CD8+ T cells, interacting with dendritic cells and keratinocytes (3), have been implicated in ACD, but current models suggest CD8+ effector T cells play a major role in the elicitation of contact allergic reactions in mice (4, 5) and probably also in humans (6). Studies in mouse models of skin sensitization have shown that strong skin sensitizers induce an oligoclonal T cell response with a high frequency of CD8 effector T cells (7). The details of the recognition by T cells of chemical allergens are still poorly understood. An exception is the response to divalent metal cations such as nickel, which is a common cause of ACD. Metal ions have been shown to bind directly to major histocompatibility complex (MHC) molecules or to peptide/MHC complexes, thus creating new antigenic determinants that are recognized as foreign by the T cell compartment (8).

As skin-sensitizing chemicals are haptens, and too small to trigger immune responses by themselves, it has been proposed that the vigor of the immune response elicited by different sensitizing chemicals (and thereby their relative potency) might correlate with chemical reactivity and their ability to form stable associations with proteins or peptides (9–11). Greater chemical reactivity has been suggested to translate into a broader range of modified peptides, creating a wider range of potential T cell epitopes and hence driving a broader and stronger T cell response (12). A global analysis of the repertoire of antigen receptors expressed on T cells that respond to a skin sensitizer may provide further insight into the processes, which drive skin sensitization and ACD. In so doing, our aim has been to develop a quantitative, mechanistic understanding of how the dose of chemical sensitizer exposure relates to the likelihood of inducing skin sensitization in humans. It is anticipated that such understanding will inform and improve the safety assessment of chemicals (2, 13).

Recent advances in massively parallel high throughput sequencing have opened the possibility of global analysis of the T cell repertoire, and several protocols have been reported. We have developed a robust single-strand DNA ligation protocol, which tags each molecule of T cell receptor (TCR) αβ messenger RNA in a sample with a unique molecular barcode before PCR amplification and sequencing (14). The most important aspect of our protocol is that it allows us to collect quantitative repertoire data that accurately reflect TCR abundance. Specifically, the molecular barcode allows us effectively to correct both for PCR bias and for sequencing error at the analysis stage (15). This is not currently possible using the commercial DNA-based TCR sequencing platforms. We have also developed a suite of software tools for the analysis of the TCR data obtained, which can be used to process the raw sequence files and assign a unique unambiguous V, J, and complementarity-determining region (CDR)3 to each sequence (16).

In the investigations described here we have applied these experimental techniques to characterize the *in vitro* response of T cells from individuals sensitized to paraphenylenediamine (PPD), a component of some hair dyes (17) and a strong skin sensitizer (18). PPD–human serum albumin (HSA) conjugate is shown to induce proliferation of a subset of peripheral blood T cells from sensitized individuals. This proliferative response is associated with increased abundance of a small proportion of TCRs, with a highly skewed V and J gene usage. The repertoire of expanded TCRs reflects antigen-driven proliferation of diverse sets of sequence-related TCRs.

## Patients and Methods

### Donor Characteristics

Patients were recruited from those attending the Contact Dermatitis Investigation Unit at Salford Royal Hospital for diagnosis (by epicutaneous patch testing) of contact allergy. Peripheral blood samples (20 ml) were drawn from patients (*n* = 56), diagnosed as being sensitized to PPD into lithium heparin-coated collection tubes (Thermo Fisher Scientific) (15 IU/ml). All patients were patch-tested to the hospital standard battery and additional relevant series and their own products using Finn Chambers^®^ on Scanpor^®^tape. Readings were undertaken at 48 h (day 2) and 96 h (day 4) according to ICDRG guidelines. Blood samples were obtained from patients who were positive to PPD and graded as + (weak; *n* = 20), ++ (strong; *n* = 29), and +++ (extreme; *n* = 7). The study was approved by NRES Ethics Committee North West, Greater Manchester East (12/NW/0602), and all patients gave written informed consent.

### Peripheral Blood Mononuclear Cell (PBMC) Isolation and Phenotyping by Flow Cytometric Analysis

Peripheral blood mononuclear cells were prepared by density gradient centrifugation with Histopaque1077 (Sigma-Aldrich). Briefly, 20 ml of whole blood was layered onto 15 ml of Histopaque1077, the samples were centrifuged at 500 × *g* for 30 min, and PBMC layers were collected and counted with a hemocytometer. An aliquot (1 × 10^6^ cells) of PBMCs from each sample was stored at −80°C in heat-inactivated human AB serum (Sigma-Aldrich) containing 10% dimethyl sulfoxide. The frequencies of different cell populations were analyzed in these previously frozen samples by the use of multiparameter flow cytometry. Surface staining with anti-human CD4–allophycocyanin (APC), anti-CD8–APC, anti-CD45RA–fluorescein, and anti-CD27–R-phycoerythrin-Cy5 antibodies was carried out in flow cytometry staining buffer for 30 min at 4°C (all reagents from eBioscience). The viability of each sample was determined by the use of forward-scattered and side-scattered light parameters for size exclusion, and an unstained control sample and appropriate isotype controls were used for setting gates. Single stains with anti-human CD3 antibodies coupled to different fluorophores were used to assess compensation settings prior to acquisition, and to establish a compensation matrix for data analysis. At least 10,000 cells were analyzed on a FACS-calibur™ flow cytometer (Becton Dickinson) by the use of FlowJo software (Tree Star). T lymphocyte subsets were identified by CD4 and CD8 expression analysis. Subsequent multiparameter quadrant analysis on these T lymphocyte populations further characterized phenotypes using CD45RA and CD27 (19). Thus, naive cells were defined as CD45RA+ CD27+, central memory (CM) cells as CD45RA− CD27+, effector memory (EM) cells as CD45RA− CD27−, and EM RA positive (EMRA) cells as CD45RA+ CD27−.

### Generation of PPD–HSA Conjugate

A solution of HSA (Sigma-Aldrich) at 10 mg/ml (150 µM) and 15 mM PPD (Sigma-Aldrich; ×100 molar excess) was dissolved in 10 ml of phosphate-buffered saline (PBS). The reaction mixture was stirred at room temperature for 48 h, and the solution was dialyzed against PBS for a further 72 h, with the PBS being changed every 4–12 h. The solution was lyophilized, and the conjugate was then stored as a powder at −80°C or dissolved in PBS and stored at −20°C for use in *in vitro* assays. The degree of HSA modification by PPD was measured by hapten–protein substitution analysis. Briefly, 200 µl of PPD–HSA or HSA at 10 mg/ml in PBS and PBS alone were incubated at room temperature for 20 min with 5 µl of 0.03 M 2,4,6-trinitrobenzenesulfonic acid (Sigma-Aldrich). The optical density was measured at 405 nm on a Biotek reader.

### [^3^H]Thymidine Proliferation Assay

The culture medium consisted of RPMI-1640 supplemented with l-glutamine (2 mM), 10% heat-inactivated human AB serum, HEPES (1%), penicillin (100 U/ml), and streptomycin (100 µg/ml) (all from Sigma-Aldrich). PBMCs were cultured in 96-well flat-bottomed plates, at 1 × 10^5^ cells/well, in 200 µl of complete medium, in the presence of PPD–HSA (0.01–100 µg/ml) or HSA only (0.01–100 µg/ml) for 6 days. Each well was pulsed at 120 h with 22.2 μBq of [^3^H]thymidine; the cells were then harvested and processed for β-scintillation counting 16 h later. A positive proliferative response was defined as one that resulted in a proliferation index of ≥2.5 as compared with control (medium only) cells. Stimulation with irrelevant antigen controls resulted in proliferation indices of <2.5.

### TCR Sequencing

RNA was isolated from 5 × 10^6^ *ex vivo* PBMCs derived from PPD-sensitized donors using an RNeasy MiniKit (Qiagen). The remaining PBMCs were cultured for 6 days in 96-well flat-bottomed plates, at 1 × 10^5^ cells/well, in 200 µl of complete medium, in the presence of 100 µg/ml PPD–HSA, 100 µg/ml HSA, or medium only. Cells from six wells per condition were pooled, and RNA was isolated using the RNeasy MicroKit (Qiagen). Up to 500 ng of RNA were DNase-treated to remove residual genomic DNA using RQ1 DNase (Promega). cDNA was generated using two TCR gene-specific primers (αRC2 and βRC2), and following the manufacturer’s instructions for SuperScript III (Invitrogen) in final volume of 30 µl with the exception that RNasin (Promega) was used instead of RNaseOUT. cDNA was purified and concentrated using MinElute columns (Qiagen). The following ligation reaction was used as part of a 5′-RACE strategy to label every cDNA molecule uniquely with a barcode (or unique molecular identifier) containing 12 random nucleotides: 10 µl cDNA, 1× T4 RNA ligase buffer (NEB), 1.5 µM BSA (NEB), 1 mM hexammine cobalt chloride, 0.33 mM ATP (NEB), 0.33 µM ligation oligo (6N_I8.1_6N_I8.1_SP2, Sigma), 10 µl of 50% PEG8000 (NEB), and 20 units T4 RNA ligase 1 (NEB) in a total reaction volume of 30 µl. Samples were incubated at 16°C for 16 h and the enzyme inactivated at 65°C for 10 min. Also, 70 µl of water were added before the reaction was purified at a 1:2 bead:sample ratio using AMPure XP SPRI beads (Beckman Coulter) according to manufacturer’s instructions and eluting in 30–35 µl water. Next, two consecutive PCR reactions were performed to amplify the samples and introduce index sequences to multiplex several samples on one sequencing run as well as introduce sequences essential for the sequencing reaction. The conditions for PCR 1 were 1× HF buffer (NEB), 0.5 µM of αRC1, βRC1.1, βRC1.2, and SP2 primers, 0.5 mM dNTPs (Life Technologies), and 1 unit Phusion polymerase (NEB). The 50 µl reactions were run on a Thermal cycler; initial cycle: 98°C, 3 min; cycle 1–4: 98°C, 15 s; 69°C, 30 s, and 72°C 40 s; final cycle: 72°C, 5 min. The PCR product was purified with AMPure beads (ratio 0.8:1 bead:sample) and eluted in 30 µl water. The second PCR was performed as a qPCR (ABI 7500), so that the reaction could be halted when enough material had been amplified. A CT threshold of 0.01 resulted in sufficient material for sequencing, and 14–18 cycles of qPCR were usually needed to reach that threshold. TCRα and β chains were amplified separately. A 25 µl reaction contained 12.4 µl sample, 1× HF buffer (NEB), Cybrgreen (Life Technologies), 0.25 µM dNTPs, Rox (Life Technologies), 0.05 µM of primers SP1-6N-I-X-αRC1 or SP1-6N-I-X-βRC1.1+1.2, SP1-P5 and P7-LX and 0.5 units Phusion (NEB). The samples were purified as before, and final products were quantified using a Qubit fluorometer (Life Technologies) and fractionated on a TapeStation (Agilent). Mixes of up to 12 samples were sequenced on an Illumina MiSeq, using version 2 chemistry 2x250PE kits.

Sequences were first demultiplexed, according to the two indices that had been introduced into each sample, using a custom Python script. A modified version of Decombinator (16) was then used to identify the V and J region used, the number of nucleotide deletions from the V and the J gene, the inserted nucleotides, and the barcode information. Finally, a custom PCR- and sequencing-error correction script was used to quantify the number of TCRs sequenced. The Python scripts are available at https://github.com/innate2adaptive/Decombinator. The raw sequence data fastq files are available at the NCBI Short Read Archive with accession numbers SAMN06270339, SAMN06270340, SAMN06270341, SAMN06270342, SAMN06270343, SAMN06270344, SAMN06270345, and SAMN06270346.

## Results

**Figure 1:**
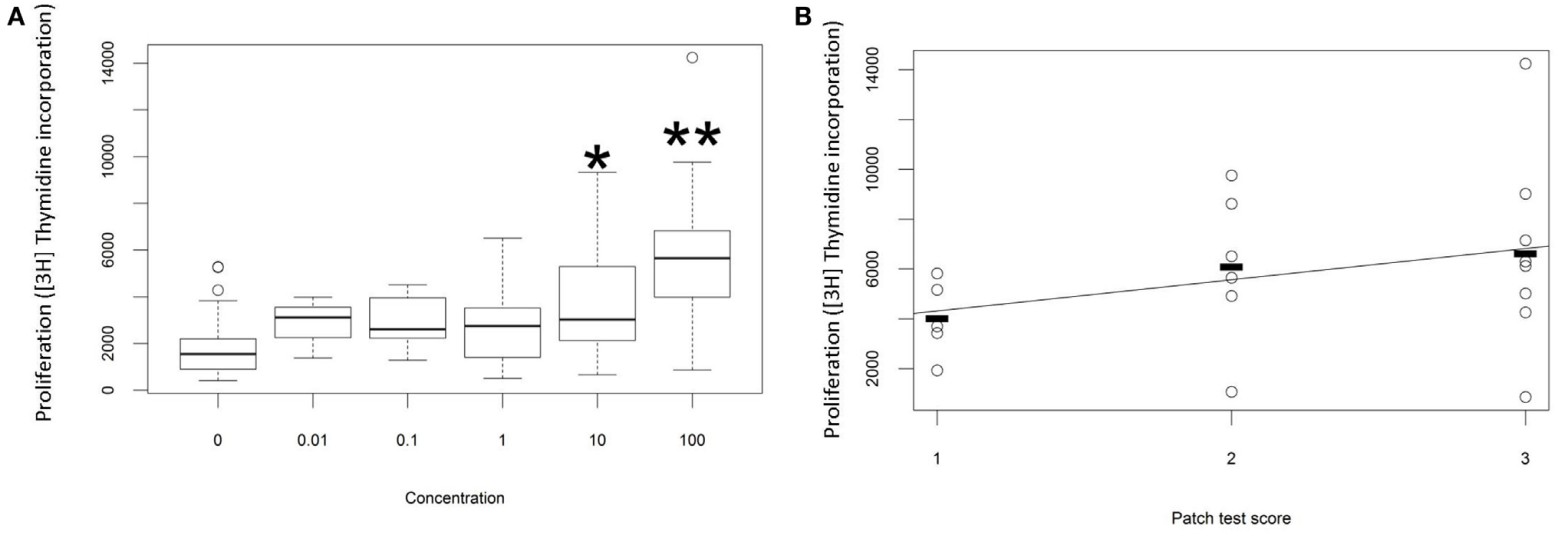
**Proliferation *in vitro* following challenge with paraphenylenediamine (PPD)–human serum albumin (HSA) conjugates**. **(A)** Median, interquartile range, and 95th percentile thymidine incorporation for all samples as a function of *in vitro* antigen dose. Stars indicate significant difference by one-way ANOVA (*p < 0.05; **p < 0.001). **(B)** Thymidine incorporation using 100 µg/ml PPD–HSA as a function of patient patch test result.

The proliferative response of peripheral blood T cells from a cohort of individuals with ACD (as diagnosed by patch test result and clinical history) to PPD was measured *in vitro*. In preliminary studies we added free PPD to the culture wells, but the chemical proved to be cytotoxic at higher concentrations, and the proliferative response was therefore difficult to quantify reproducibly. Instead, we made conjugates of PPD and HSA and measured the response to the chemical conjugate. As described previously (20), we observed a dose-dependent proliferative response to PPD–HSA, with significantly increased [^3^H]thymidine incorporation at 10 and 100 µg/ml conjugate (Figure 1A). Further experiments used 100 µg/ml as this gave the maximum proliferation. The proliferation showed a trend to correlation with patch test score (Figure 1B) although there was substantial variability in the proliferation observed in patients with high score, such that the correlation did not reach statistical significance with this number of patients.

**Figure 2:**
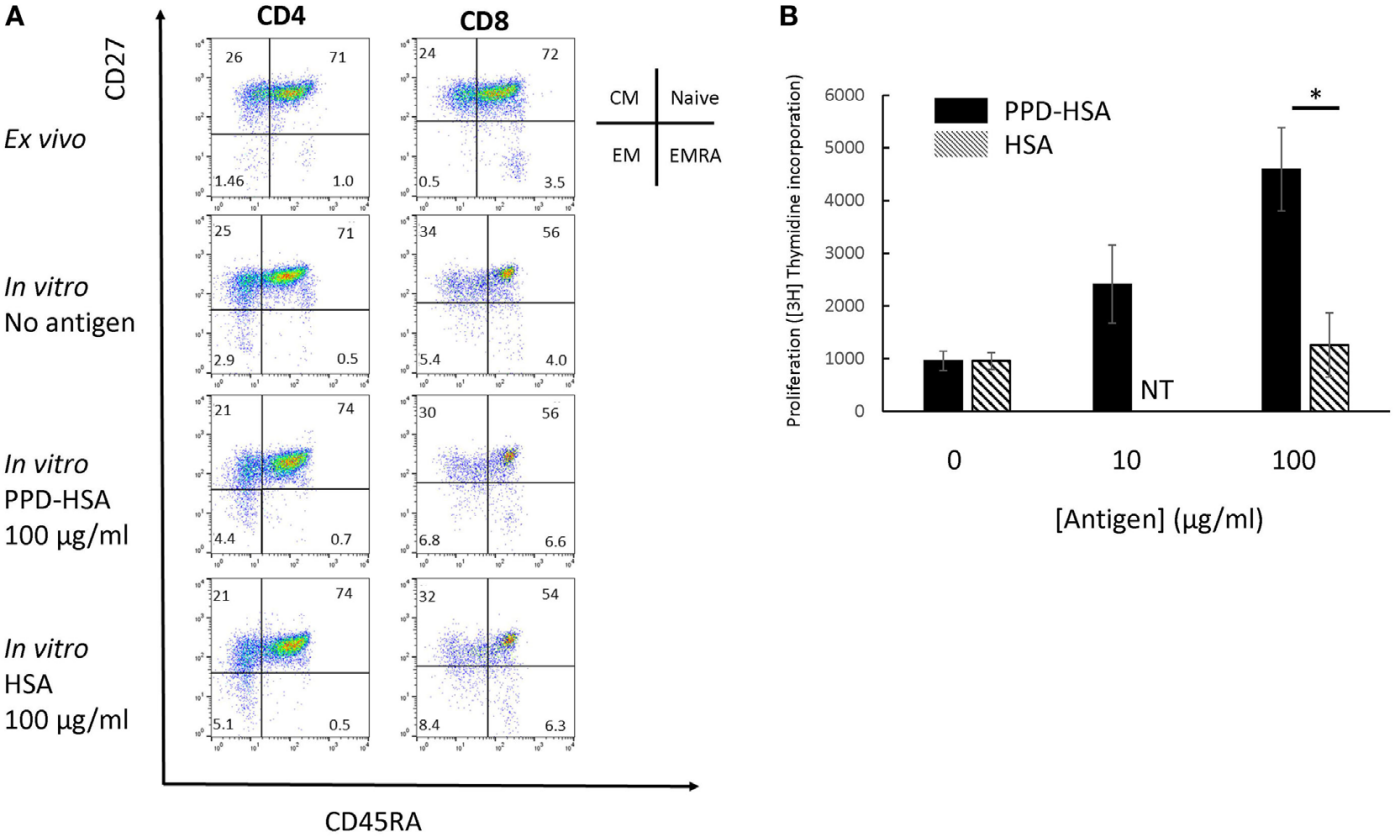
**Memory phenotype and thymidine proliferation for patient 91**. **(A)** Flow cytometry showing the four populations of naïve, central memory, EM and EMRA as defined by the combination of CD45RA and CD27 expression for CD4 (left) and CD8 (right). The top row shows phenotype of cells directly *ex vivo*, while the lower two rows show the phenotype after *in vitro* culture with or without antigen as shown. **(B)** Proliferation (thymidine incorporation) in response to paraphenylenediamine (PPD)–human serum albumin (HSA) (filled) or HSA (mean and SD, 4 replicate cultures per point). NT, not tested. *p < 0.05 (Student’s *t*-test).

We looked in further detail at the phenotypic changes observed in the proliferation cultures. The flow cytometry results on a typical sample with high *in vitro* antigen-specific proliferation (1 of 12 independent experiments analyzed) is shown in Figure 2A, and the accompanying proliferation to PPD–HSA and to the HSA control are shown in Figure 2B. The CD4 and CD8 cells were analyzed separately and stained with a combination of CD27 and CD45RA to define naive, CM, EM, and EM CD45RA revertants (EMRA) (19). The *in vitro* culture contained a smaller proportion of fewer CD8+ naïve cells (perhaps because of selective cell death). The addition of antigen (PPD–HSA, 100 µg/ml) caused a small shift from CM to EM in both CD4+ and CD8+ populations, when compared to cells cultured in medium alone. This effect albeit small was seen consistently. The mean decrease in CM CD4+ was 9% (SEM = 2.5, *n* = 5), and the corresponding increase in EM CD4+ was 9 (SEM = 2.6, *n* = 5). The mean decrease in CM CD8+ was 2% (SEM = 1.0, *n* = 5), and the corresponding increase in EM CD4+ was 2% (SEM = 0.8, *n* = 5). HSA alone caused similar phenotypic changes, suggesting some non-specific carrier-dependent effect. However, thymidine incorporation indicates less cell proliferation in response to HSA, suggesting that HSA may induce phenotypic maturation with less proliferation than PPD–HSA.

Interestingly, we noted that the *ex vivo* CD4 and CD8 populations showed a profound depletion in the proportion of EM populations *in vivo*, compared to the values reported previously (19). This observation was consistent among different patients and replicates an observation made previously on PBMC from patients with allergy to methylisothiazolinone (21). Since we did not have access in this study to blood samples taken before the patch test, we could not determine whether the observation related to allergic status or as a response to the exposure to PPD in the diagnostic patch test, so this observation was not pursued further.

**Figure 3:**
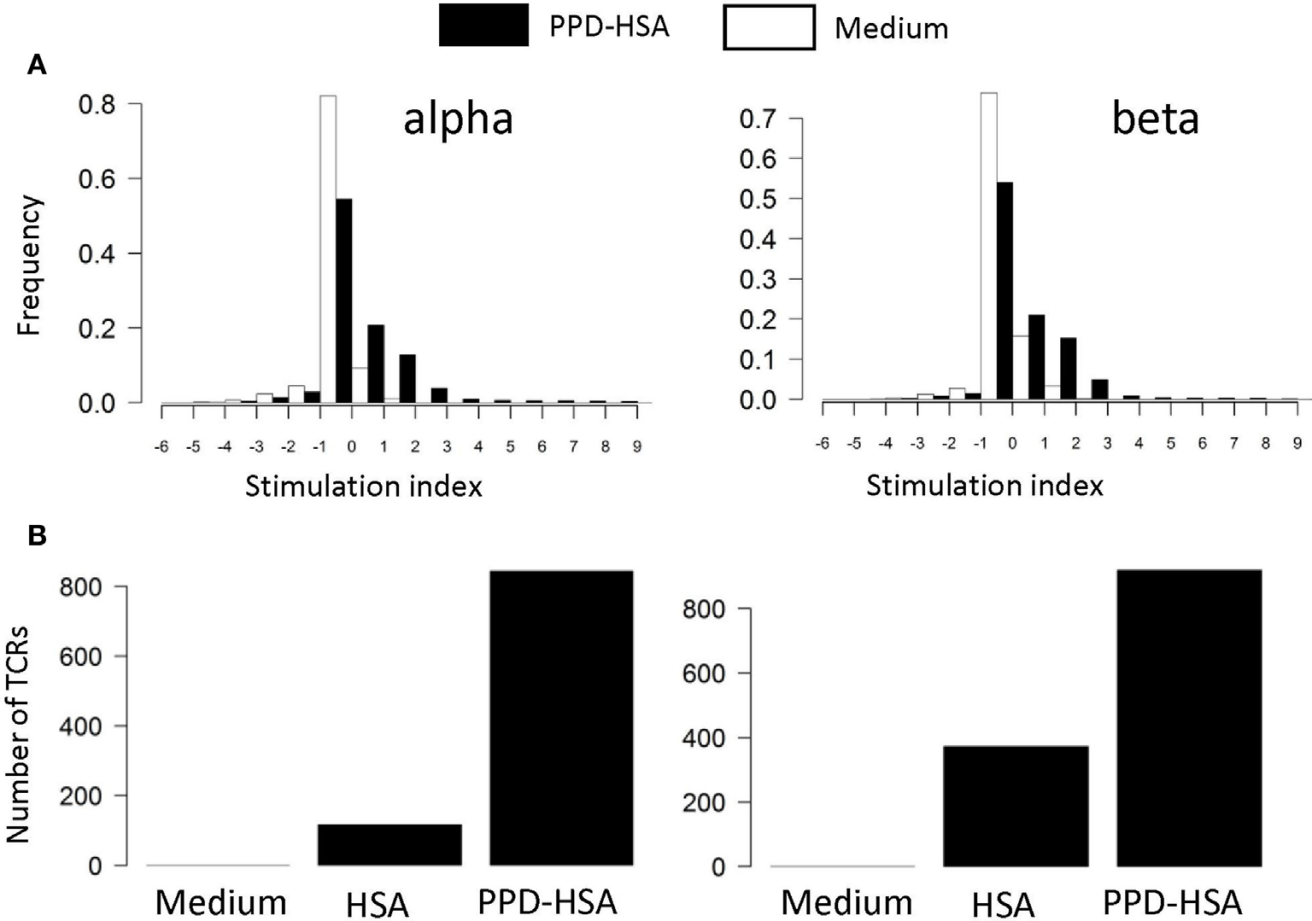
**(A)** Stimulation index (SI) distribution for *in vitro* T cell receptors (TCRs). The SI is calculated as the logarithm (base 2) of the abundance in the *in vitro* culture with medium alone (empty bars) or stimulated with 100 µg/ml paraphenylenediamine (PPD)–human serum albumin (HSA) (filled bars) relative to the abundance in the *ex vivo* sample. The abundance of the TCRs that are absent from the *ex vivo* sample is estimated as 1 (mean abundance in *ex vivo* samples is 1.3). **(B)** The number of TCRs with a SI > 3 (i.e., more than eightfold expanded in *in vitro* culture) for cultures in medium, HSA, or PPD–HSA.

We next examined the TCR repertoire of the T cells cultured *in vitro*. The T cells were collected after culture, pooled to give 0.5–1 × 10^6^ T cells, and processed for TCRα and TCRβ sequencing. The pipeline generates a list of distinct TCR sequences for each sample, together with an abundance indicating the number of times the sequence is found within that sample. The abundance of each distinct TCR found in the *ex vivo* and in the *in vitro* stimulated samples (HSA or PPD–HSA) was plotted. We find a clear expansion of TCRα and β sequences following PPD–HSA stimulation that do not expand following HSA stimulation but are present at lower frequency in the HSA only as well as in the *ex vivo* sample. For each distinct TCR sequence identified after 6 days of culture, we then calculated a stimulation index (SI), defined as the logarithm (base 2, equivalent to the number of cell doublings if cell death is ignored) of the abundance in the *in vitro* culture relative to the abundance in the *ex vivo* sample. The abundance of the TCRs that were absent from the *ex vivo* sample (due to sampling) is estimated as 1 (since abundances of *ex vivo* samples have a mean of 1.3, median of 1, and TCRs observed *in vitro* after proliferation must have been present at the start of the proliferation assay with an abundance of at least 1). The SI for cultures with medium alone or in the presence of antigen (PPD–HSA, 100 µg/ml) is shown in Figure 3A. The majority of TCRs show no change or a slight decrease in frequency following *in vitro* culture. This is consistent with the overall stability of phenotype seen after culture *in vitro*. However, a small number of sequences are found at much higher abundance after culture in the presence of antigen. The number of distinct TCRs with an SI greater than 3 (eightfold increase or three divisions) in cultures with medium alone, PPD–HSA, or HSA alone is shown in Figure 3B. The qualitative pattern observed is similar to the pattern of thymidine incorporation shown in Figure 2B. The approximately 800 expanded TCRα and TCRβ sequences are equivalent to about 0.8% of the total number of TCRs detected, reflecting the specificity of the proliferative response observed.

**Figure 4:**
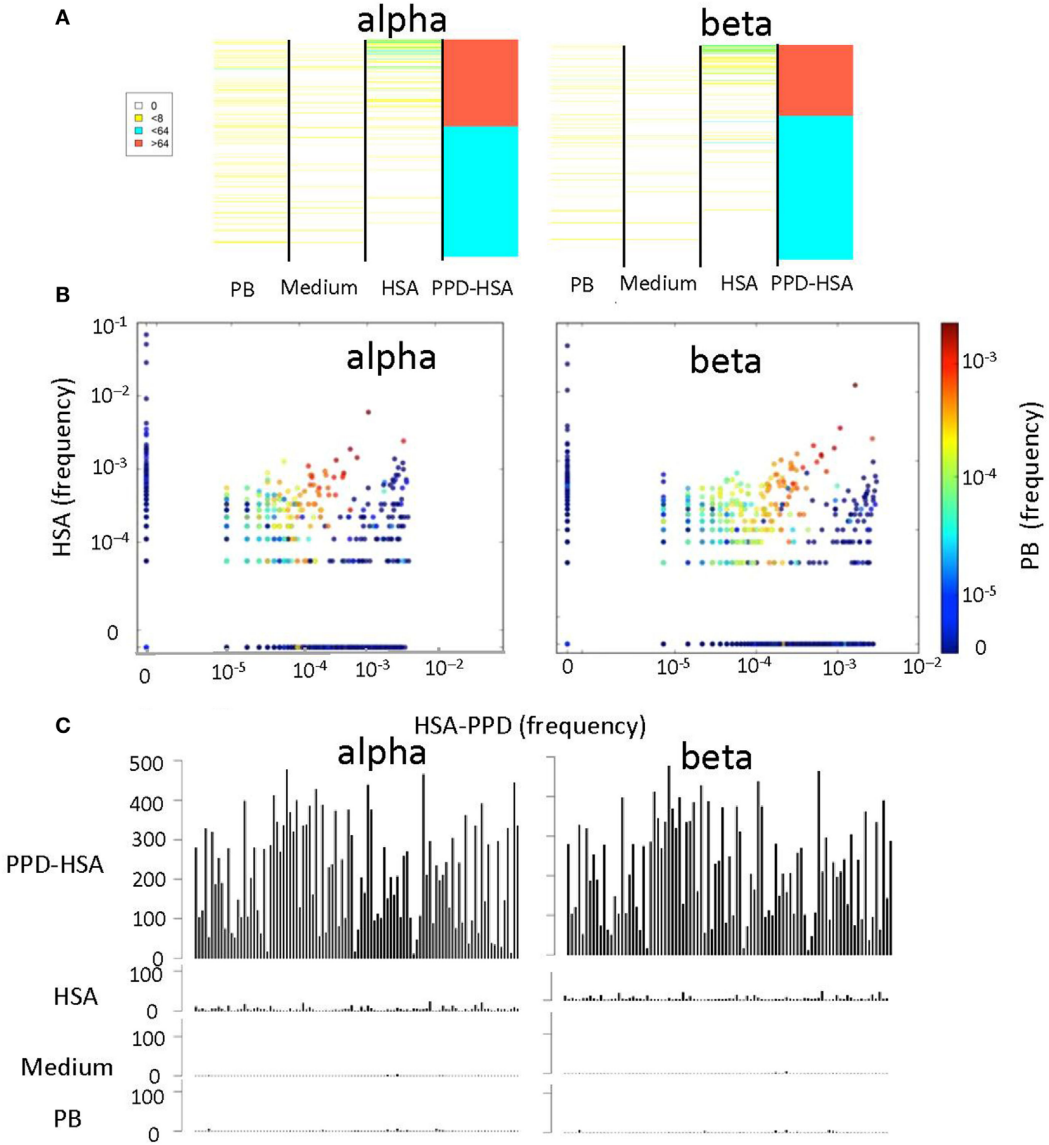
**The relative T cell receptor (TCR) abundances in different *in vitro* cultures**. **(A)** Heat map showing the abundance of each TCR with a stimulation index (SI) > 8 in paraphenylenediamine (PPD)–human serum albumin (HSA) cultures, in cultures with medium alone, HSA, or in the *ex vivo* (PB) sample. Each row of the heat map is a distinct TCR gene sequence, colored according to its abundance. The TCRs are ranked in order of their abundance in the PPD–HSA cultures. **(B)** The relative frequency of TCR in PPD–HSA (*x* axis) cultures versus HSA alone (*y* axis). Each dot represents a distinct TCR, and the color represents frequency in the PB sample. **(C)** Histograms of the abundances in all four repertoires of all TCRs with an SI > 8 in PPD–HSA cultures and an SI > 1 in HSA cultures.

We next compared the abundance (Figure 4A) or the frequency (Figure 4B) of the TCRs that were expanded in the PPD–HSA cultures, in the HSA only, medium only (in Figure 4A only), or *ex vivo* repertoire. In general, the TCRs expanded in PPD–HSA were not observed at high abundance in the other cultures. However, in many cases, the sequence was simply not observed in the corresponding other cultures. This could have been due to differential amplification or sampling (i.e., the relevant precursor was not present in the culture at the start). In order to look more carefully at specificity, we plotted the abundance of all the clones shown in Figure 4A, which were also present at least once in the HSA culture (Figure 4C). The abundance of the TCRs varied quite broadly, with some TCRs showing abundances of >500 after culture in PPD–HSA. In contrast, abundances in the HSA cultures were always less than 10, suggesting that the expansion observed was specific to the presence of the PPD hapten.

**Figure 5:**
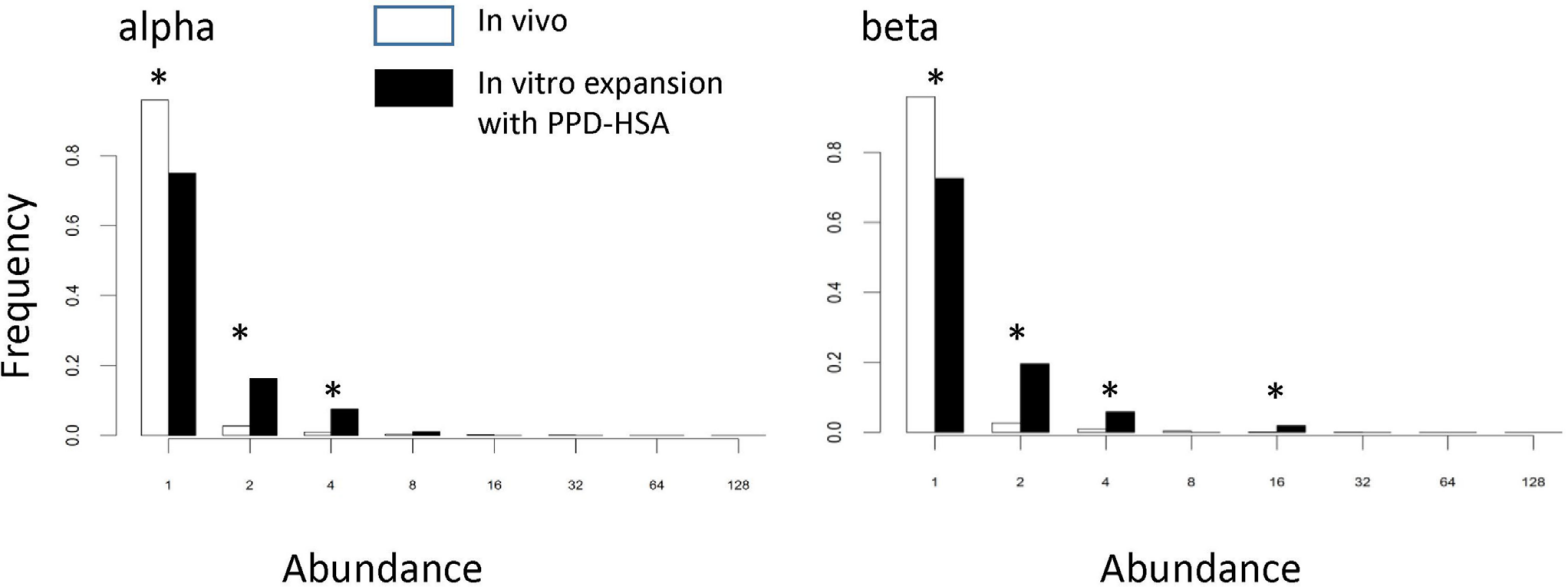
**The abundance distribution of all complementarity-determining region (CDR)3 in the *ex vivo* sample (empty bars) compared to the abundance of T cell receptors (TCRs) in the *ex vivo* samples that are expanded following paraphenylenediamine (PPD)–human serum albumin (HSA) stimulation *in vitro* (the PPD–HSA expanded) [stimulation index (SI) > 3] TCRs (filled bars)**. Asterisk shows those values for which the proportion differs significantly (*p* < 0.01) using the *z*-test for proportions.

We examined whether the TCRs that had expanded in the presence of PPD–HSA (SI > 3) represented a population, which had been pre-expanded *in vivo*. Figure 5 shows the abundance of the PPD–HSA expanded TCRs in the *ex vivo* pre-expansion sample. The distribution is compared to the overall abundance distribution in the *ex vivo* sample from the same donor. The histogram for the expanded set of TCRs shows a clear shift toward higher abundance, with >20% of the expanded TCRs having an abundance of >2 in the PBMC sample. Thus the set of TCRs, which expand *in vitro* are already present at higher frequencies than average *in vivo*, consistent with being derived from an allergen-induced memory T cell response.

**Figure 6:**
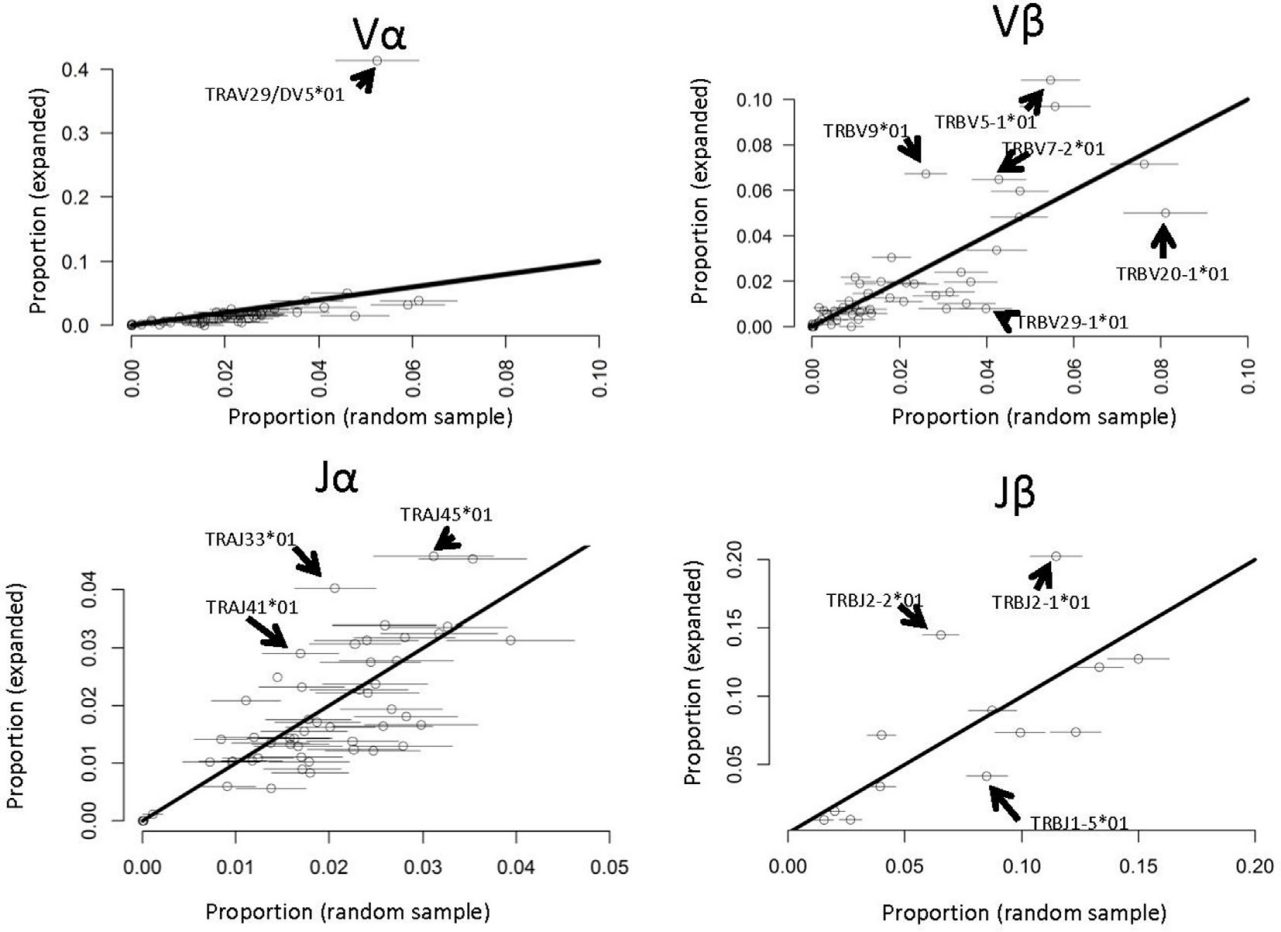
**V and J gene usage in paraphenylenediamine–human serum albumin expanded T cell receptors (TCRs) compared to random TCRs**. For each V or J gene, the abundance in the expanded TCR set illustrated in Figure 4A is shown along the *y* axis, and the abundance (mean plus 2 SDs) in 100 equal-sized random samples of TCRs drawn from the *ex vivo* repertoires is shown along the *x* axis. The full names of those genes, which differ significantly from the diagonal (i.e., are over- or underrepresented in the expanded set) are shown.

Several previous studies have reported that antigen-specific responses often favor specific V genes [e.g., Ref. (22)]. We therefore compared the frequency of each V and J α and β gene in the PPD–HSA expanded TCRs with their frequency in the *ex vivo* pre-culture sample (Figure 6). Since the size of the set of expanded TCRs is rather small (in the order of 800) we selected 100 repeated samples from the *ex vivo* repertoire to estimate sampling heterogeneity. We then plotted the frequency in the antigen-expanded sample with the frequency in blood. The majority of genes fell in or near the diagonal, indicating a similar frequency in PBMC and in antigen cultures. However, for both V and J genes, one or more gene showed substantial changes after culture. TRAV29/DV5, for example, was present at 10 times its frequency in the matched PBMC sample (Figure 6). Thus *in vitro* culture with PPD–HSA conjugate drives selective expansion of TCRs with skewed V and J gene usage.

**Figure 7:**
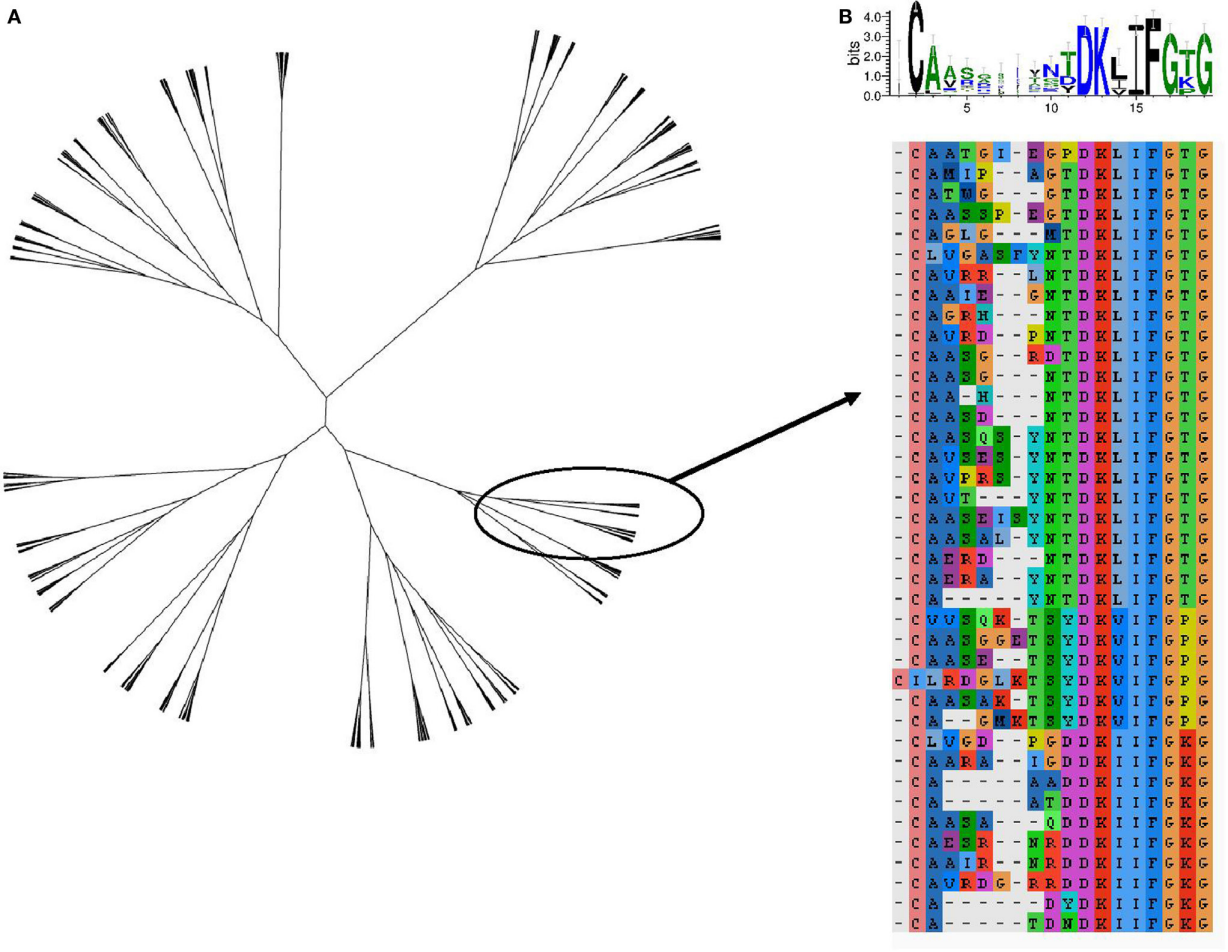
**The sequence relationship between the complementarity-determining region (CDR3)β sequences of the T cell receptors (TCRs), which are expanded [stimulation index (SI) > 3] after culture in the presence of paraphenylenediamine–human serum albumin**. **(A)** Unrooted tree of all expanded TCRβ CDR3 clustered according to Levenshtein distances (Ward agglomerative clustering). **(B)** Multiple sequence alignment and sequence logo of the subtree of **(A)** containing the most expanded CDR3β sequence.

The TCR region with the greatest contact with antigen/MHC complex is the CDR3 loop. The relationship between two CDR3 sequences can be captured by the Levenshtein distance, the number of changes (i.e., substitution, deletion, and addition) required to transform one CDR3 into the other. The amino acid sequences of the 800 expanded TCRs were clustered according to their Levenshtein distance matrix (Figure 7A). The sequences fall into a number of families of closely related sequences. A multisequence alignment of the subtree containing the most abundant TCRβ CDR3 member is illustrated as an example (Figure 7B). The sequences within a family show a high degree of conservation, especially toward the N terminal part of the sequence. However, the overall average Levenshtein distance between all expanded CDRs (α = 9.53, β = 8.61) is only slightly smaller than between a random sample of *ex vivo* (pre-expanded) TCRS (α = 9.81, β = 9.47). PPD–HSA exposure therefore drives the expansion of families of TCRs, which are strongly conserved within a family but weakly related or unrelated between families.

## Discussion

Paraphenylenediamine is widely used as a hair dye (17) and as a component of black henna tattoos (23), and the overall prevalence of PPD contact allergy was 0.8% in a recent study of European populations (18). The immune response to PPD, therefore, serves as a good model with which to study the immune response to contact allergens and to understand better the nature of allergen recognition. In this study we analyzed the T cell response to PPD at the level of TCR repertoire, highlighting the breadth of TCRs associated with antigen-induced proliferation.

The response to PPD, and to contact allergens in general, is believed to reflect a T cell response, comprising both CD4+ and CD8+ T cells (1). We therefore measured T cell *in vitro* proliferative responses in PBMC from a cohort of individuals clinically diagnosed as PPD-allergic and who were shown to be patch test positive. A chemical conjugate of PPD with HSA was used as it is less cytotoxic than free PPD and has previously been characterized in detail (20, 24). We first confirmed previous reports that peripheral blood T cells show a dose-dependent antigen-specific response to PPD–HSA, as measured by [^3^H]thymidine incorporation. *In vitro* culture was associated with a selective loss of naïve T cells and a small shift from CM to EM in cultures exposed to antigen.

Interestingly, we noted that the starting PBMC population from the PPD-allergic individuals appeared to be depleted of EM cells, compared to the values reported previously for healthy volunteers (19). This might reflect recruitment of effector cells from blood to the skin either due to the patch test carried out 4 days prior to venepuncture or to the allergic condition itself. A similar observation was recently reported for another skin sensitizer, methylisothiazolinone (21). Further longitudinal studies will be required to establish the cause of this relative paucity of EM cells in the peripheral blood and its possible impact on responses measured *in vitro*.

Our TCRseq protocol incorporates unique molecular identifiers, which allow us to count accurately the number of TCR RNA molecules present in a sample. The relationship between RNA count and cell count depends on the number of TCR RNA molecules per cell. Although we cannot rule out some changes in RNA levels during different phases of T cell activation, previous studies do not suggest substantial changes (25, 26) and would not contribute significantly to the large changes in abundance we observe.

An SI of approximately 9, equivalent to a 500-fold increase in abundance, is consistent with a culture period of 6 days, and T cell replication times of around 24 h for the first generation, and 16–18 h for subsequent generations (27). We do not have dynamic information on T cell numbers during the culture, but since thymidine incorporation is a function of those cells still in S phase during the last day of culture, these results suggest that the observed proliferation can be attributed to a few thousand cells derived from only 2–4 precursors per 100,000 T cells. These TCRs are not expanded in cultures without PPD–HSA, suggesting they represent antigen-specific responses to the PPD conjugate.

Together with a very small number of T cells that continue to proliferate throughout the culture, we also observed many more cells (approximately 0.8% of total), which increase in abundance by a smaller amount. We cannot determine whether these cells represent PPD-specific cells of lower affinity, which respond less robustly to antigen, or perhaps include bystander cells activated *in vitro* as a result of antigen-presenting cell activation and cytokine secretion.

The sequences of the TCRs expanded *in vitro* show hallmarks of an antigen-specific response. They exhibit strong skewing of both V and J gene usage (28–30), and they consist of families of TCRs with conserved α and β CDR3s. Nevertheless, the *in vitro* expanded TCRs show only slightly greater similarity to each other than they do to a random selection of CDR3s from the *in vivo* repertoire, and the clones showing a high SI are found in several different and quite distinct families of CDR3 sequences.

It is instructive to compare the results observed here with those of an extended series of studies on T cell responses to another strong contact sensitizer, trinitrophenyl (TNP) sulfonic acid [reviewed in Ref. (31)]. Although carried out prior to the era of high throughput sequencing, and in an experimental mouse model, these studies reported remarkably similar qualitative features of the contact sensitizer-specific responses. In particular, the TNP response was associated with a high frequency of hapten-specific T cells (32), was associated with strong preferential usage of certain V and J regions in both alpha and beta chain (33, 34), but was nevertheless very polyclonal involving a wide variety of different TCR sequences (9, 35). These features may therefore represent common features of the sensitizer-specific repertoire in both mouse and man.

The results of our study are consistent with a model in which PPD–HSA conjugate stimulates a broad diversity of TCRs, and with a wide range of stimulation strengths, which manifest as different degrees of *in vitro* expansion. The reasons for the breadth of response observed remain to be defined. Although PPD has been shown to react principally with a single free cysteine in HSA (20), smaller amounts of alternative conjugate products may also be present, giving rise to a variety of hapten/protein conjugate peptides. Alternatively, a single PPD–HSA-derived peptide may be processed and presented by APC in a variety of ways and recognized by a variety of T cells.

The study begins to characterize the magnitude and breadth of the T cell response to a well-characterized strong skin sensitizer, PPD (36). Further investigations are in progress to separate the contribution of CD4 and CD8 cells and to define the antigen specificity of the responding cells in more detail. The parameters learnt from such studies will help achieve a better understanding of the fundamental characteristics of the T cell response to contact allergens that can in turn be used to improve the clinical relevance of human health risk assessment for chemical skin sensitizers (2, 13).

## Author Contributions

TO and AP carried out all the T cell culture and T cell receptor sequencing. JW and NG organized patient sample collection and ethical approval for the study. KB, JH, and MI performed the analysis of the T cell receptor sequence data. RD designed the T cell phenotype studies and contributed to the manuscript. GM, IK, and BC designed and supervised the study and wrote the manuscript.

## Conflict of Interest Statement

GM and NG are employees of Unilever PLC, which funded the study.
